# Computer-based teaching is as good as face to face lecture-based teaching of evidence based medicine: a randomised controlled trial

**DOI:** 10.1186/1472-6920-7-23

**Published:** 2007-07-20

**Authors:** James Davis, Evi Chryssafidou, Javier Zamora, David Davies, Khalid Khan, Arri Coomarasamy

**Affiliations:** 1 The Education Resource Centre, The Birmingham Women's Hospital NHS Trust, Edgbaston, Birmingham, B15 2TG, UK; 2 The Medical Education Unit, The University of Birmingham, Edgbaston, Birmingham, B15 2TT, UK; 3 Clinical Biostatistics Unit, Hospital Ramon y Cajal, Ctra. Colmenar km 9.100, 2803 Madrid, Spain

## Abstract

**Background:**

At postgraduate level evidence based medicine (EBM) is currently taught through tutor based lectures. Computer based sessions fit around doctors' workloads, and standardise the quality of educational provision. There have been no randomized controlled trials comparing computer based sessions with traditional lectures at postgraduate level within medicine.

**Methods:**

This was a randomised controlled trial involving six postgraduate education centres in the West Midlands, U.K. Fifty five newly qualified foundation year one doctors (U.S internship equivalent) were randomised to either computer based sessions or an equivalent lecture in EBM and systematic reviews. The change from pre to post-intervention score was measured using a validated questionnaire assessing knowledge (primary outcome) and attitudes (secondary outcome).

**Results:**

Both groups were similar at baseline. Participants' improvement in knowledge in the computer based group was equivalent to the lecture based group (gain in score: 2.1 [S.D = 2.0] versus 1.9 [S.D = 2.4]; ANCOVA p = 0.078). Attitudinal gains were similar in both groups.

**Conclusion:**

On the basis of our findings we feel computer based teaching and learning is as effective as typical lecture based teaching sessions for educating postgraduates in EBM and systematic reviews.

## Background

Lecture based teaching is common in postgraduate and continuing education. Computer based teaching sessions have advantages over lectures: they are more flexible for doctors to fit into their work and learning programme; there is the ability to pause or revisit areas of the session; they have more learner led interaction; hyperlinks and additional materials can be provided instantly for the learner; they address the issue of standardizing the quality of teaching materials across a region; and they deal with the cost and logistical difficulties of specialist lecturers teaching large numbers of students in different locations [1,2]. Existing studies of knowledge and attitudinal gain by computer based teaching have mainly been at undergraduate level [3-5].

Education of undergraduate medical students can be enhanced through the use of computer assisted learning. Educationally it would be a mistake to apply these findings to postgraduates. Many trainers mistakenly believe that the two groups learn in the same way but this is not true. Adult learning theory suggests the determinants of learning in the two groups are different [12]. Postgraduate learning is driven by self motivation and relevance to clinical practice whereas undergraduate learning is generally driven by external factors such as curriculum and examinations [12]. The two groups therefore may react differently to new teaching interventions, such as computer based teaching. Based on adult learning theory internally driven postgraduates may be more likely to adopt computer based teaching more avidly than their undergraduate counterparts who are less worried how they are taught as long as their teaching is assessment focused.

For many educationalists it is perhaps a forgone conclusion that doctors will in the future do the majority of their learning through computers. Within dentistry and other allied healthcare groups there have been trials comparing different computer based learning educational interventions and other non computer based learning formats, [6-10]. Unfortunately there have been no randomised controlled trails at postgraduate level within medicine. Work is therefore necessary in the field of postgraduate medical education to provide evidence that moving across to computer based teaching will not lead to reduced quality teaching and learning experiences for our junior doctors.

Randomised controlled trials can provide robust evidence of educational effectiveness [11]. Randomised trials, in education could suffer due to difficulty with standardising the educational intervention(s), contamination between the two arms of a study, inability to blind the study participants and the teachers from the educational intervention(s) leading to selective co intervention, and finally difficulty with measuring outcomes due to the lack of valid and reliable assessment tools. Some of these factors make randomised trials unfeasible in educational settings, thus necessitating other designs such as non-randomised controlled and before and after studies. Despite such difficulties randomised trials have been conducted into educational interventions with some success [12].

We carried out a randomised controlled trial comparing two methods of teaching, designed in such a way so as to avoid the problems outlined above. EBM was chosen as the teaching topic as this an important area in clinical medicine [13], in medical education evidence based practice is considered a mandatory competency for postgraduates [14], and it can be taught using computer based sessions or lectures. We hypothesized that both teaching strategies will be equally effective in increasing students' learning.

## Methods

We conducted an individual randomised trial to assess the effect of teaching using self administered questionnaires before-and-after the intervention. The trial was carried out during July 2005. Exemption from obtaining ethical approval was granted by the Local and Regional Ethics Committee. The study was approved by the West Midlands Deanery. The trial compared a short computer based session with an equivalent lecture, of similar content, structure and duration, for their educational effectiveness. The intervention was CD-ROM based, but was developed in a format that could be directly uploaded onto the internet. The teaching package was developed in conjunction with the University of Birmingham's information technology department. The cost in producing the package was minimal, well within normal departmental budgets for teaching postgraduate doctors. Microsoft™ producer was used as Microsoft™ is currently the platform of most NHS operating systems and is freely available.

All postgraduate centres in the U.K. West Midlands participating in the foundation program for newly qualified doctors were invited to participate in the trial. Six centres with adequate computer facilities were included. Postgraduate trainees in these centres were randomised to either computer based session or lecture using sealed envelopes prepared by the Birmingham clinical trials unit. The randomisation sequence was generated by computer and the envelopes were coded by a third party to ensure concealment of randomisation. The format of sessions at each centre consisted of: baseline questionnaire (with the initial pretest), randomization of doctors, and simultaneous administration of educational interventions in separate areas to prevent contamination followed by post intervention questionnaire. The time allocated for teaching was forty minutes and ten minutes for each questionnaire.

**Figure 4:**
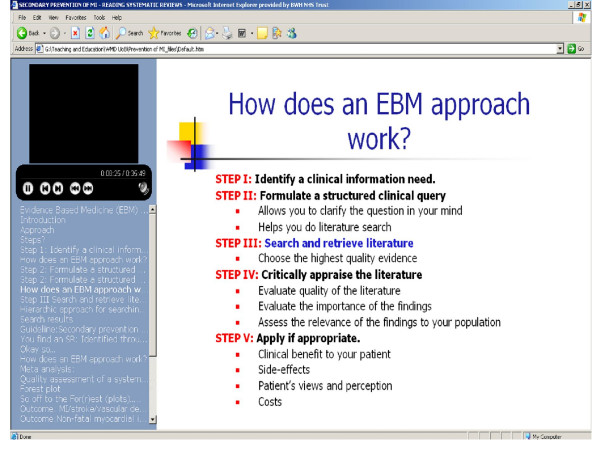
Screenshot from the Evidence based medicine teaching trail.

The interventions consisted of: (A) Computer based session; and (B) Lecture based session. The content covered EBM teaching on question framing, literature searching, critical appraisal of systematic reviews and meta-analysis, and application of findings of systematic reviews. The lecture was scripted and then recorded for the computer based version. The recording was then merged with power-point slides and links using Microsoft™ producer. The computer based teaching consisted of slides, a talking head which guided the lecture, play, pause and skip options and hyperlinks to main sections within the session, this can be seen in the screenshot (see figure 4). This was delivered by uploaded CD-ROM to participants in hospital computer clusters using individual personal computers and headphones. The lecture based session consisted of exactly the same scripted material, delivered using a session plan by the same tutor, at all the centers. Every possible effort was made to ensure the lesson plans and educational content were equivalent in the two groups; the only differences related to the method of delivery.

We developed a questionnaire for pre and post intervention measurements in knowledge and attitudes using previously validated assessment tools for evaluating EBM teaching [15-17]. From previous studies the questions we used were shown to have face and concurrent validity. The web sites linking to these questionnaires can be found in appendix 1 (see Additional file 1). Items included for knowledge assessment (primary outcome) were carefully chosen and adapted so as to achieve content validity. These were five knowledge questions (two structured questions and three multiple choice questions) with a pre-determined validated marking scheme, with a maximum score of 16. There were six attitudinal questions, previously validated for content validity, on a five point Likert scale [18]. The questionnaires were marked by an examiner blind to group allocation.

Our hypothesis was that the ability of both interventions to change students' scores would be similar. Thus we defined the primary outcome to be the change (improvement) between baseline and post intervention knowledge assessments. We predefined the range of equivalence between both arms of the trial as any difference between both groups in this variable lying within 10% of the maximum score (± 1.6 points). Assuming that the standard deviation (S.D.) of the change will be 2 points, we needed 25 subjects in each group to have 80% power with a 5% type I error rate to exclude a difference between both groups greater than the equivalence threshold. We used ANCOVA to compare score changes in the two study groups, with teaching interventions as the main factor and baseline score as a covariate [19]. To estimate the difference between intervention groups, difference between least-squares means and corresponding 95% CI were calculated based on the ANCOVA model. For ordinal data (Likert scales), non parametric statistics were used. To compare the proportion of participants with attitudinal gain we used Fisher exact tests. All statistical tests were two-sided with a significance level of p < 0.05. Analyses were done using SPSS version. 12.0 (SPSS Inc.)

The sponsor (West Midlands Deanery) had no input in conduct, analysis or interpretation of the study.

## Results

**Figure 1:**
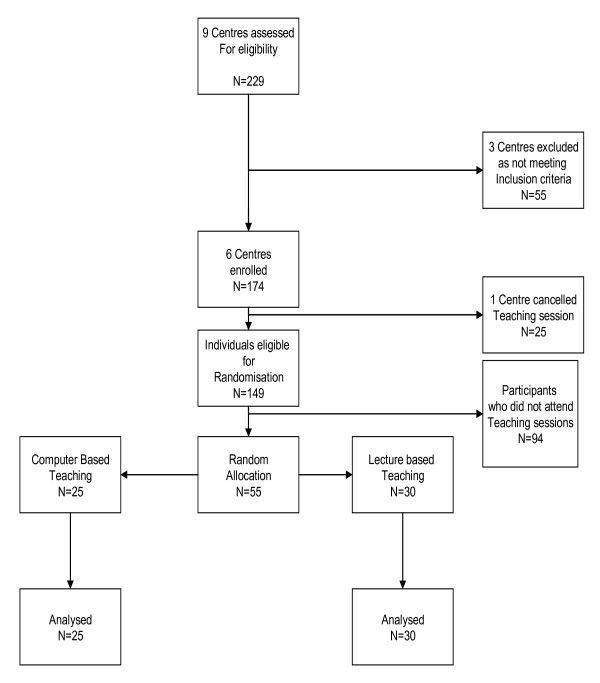
Flowchart of participants in the Evidence Based Medicine Trial.

The flow of participants throughout the trial is shown in figure 1. The baseline characteristics of the two groups are shown below in table 1. The three centres which did not meet the inclusion criteria were excluded because at the date of teaching none of these centres had adequate computer facilities to allow the trial to be run.

**Table 1:** Baseline characteristics of participants in Evidence Based Medicine teaching trial.

	Computer N = 25	Lecture N = 30
	n %	n (%)

Access to a staffed medical/health care library	25 (100)	28 (93)
Access to literature via the Internet	24 (96)	29 (97)
Searched the literature for evidence	23 (92)	22 (73)
Education or training in Research methods	11 (56)	16 (53)
Education or training in Epidemiology	9 (36)	18 (60)
Education or training in Statistics	9 (36)	17 (57)
Personally been involved in conducting research	20 (80)	20 (67)

Evidence based medicine and literature review teaching is not currently part of the UK undergraduate medical curriculum.

**Figure 2:**
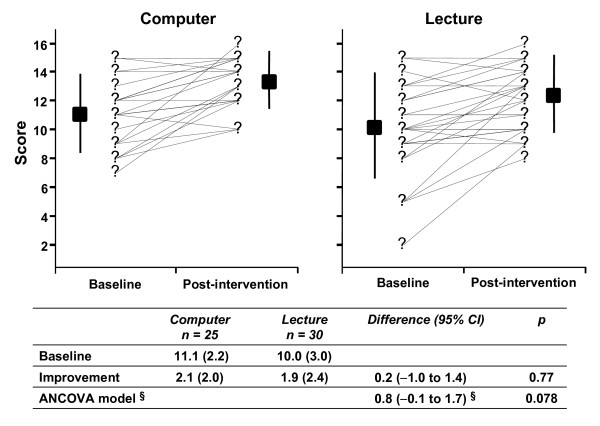
**Comparison of knowledge scores achieved through computer-based session versus lecture**. §Difference in least squares means (ANCOVA model). There were no differences between interventions at baseline

Assessments done immediately after the interventions revealed that both groups significantly improved their scores as shown in figure 2. The changes in score in the computer based teaching group and the lecture based teaching group were 2.1 [S.D. 2.0] and 1.9 [S.D. 2.4] respectively. The difference between the least-squares means computed by ANCOVA model was 0.8 (95% CI -0.1 to 1.7) and not statistically significant (p = 0.078). The confidence interval of the difference completely excludes the lower margin of inferiority of computer based sessions pre specified (methods section). It cannot exclude at the upper margin the possibility that performance of computer based teaching was slightly better than lecture based teaching.

**Figure 3:**
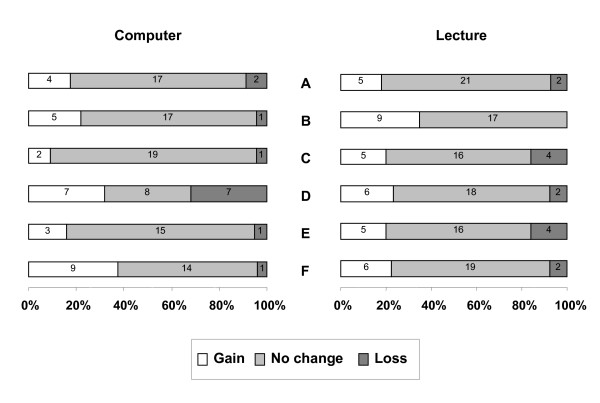
**Comparison of attitudinal gains achieved through computer-based session versus lecture**. The questions used to assess attitudes were as follows: A. EBM is a passing fashion B. Systematic reviews play a key role in informing evidence-based decision-making. C. Clinical judgement is more important than EBM. D. Systematic reviews are key to informing EBM. E. Evidence-based decision-making is "health care by numbers". F. Study design is important in article selection. Responses were measured on a five points Likert scale. Attitudinal change was defined as the change between baseline and post intervention assessments. There was no statistically significant difference between the attitudinal gains achieved through two teaching strategies (Fisher exact tests).

Comparison of attitudinal gains between the two groups showed a similar change between baseline and post-intervention (figure 3).

## Discussion

This trial showed that in our setting when teaching EBM and systematic reviews to postgraduates there was no difference between computer based session and lecture considering knowledge. We cannot exclude the possibility that computer based teaching may be better, but we are certain that it is not inferior to lectures. As a secondary outcome attitudinal gains were equivalent.

Our study represents the first randomised trial of its kind in postgraduate medical education. We were able to comply with CONSORT [20] guidelines for reporting. There was concealment of randomisation, all interventions were delivered by the same tutor, there was no contamination of interventions, the assessment was validated and blinded, and the power was sufficient to demonstrate non-inferiority. The attendance was poor so many potentially eligible doctors did not participate. Junior doctors are currently under increasing pressure to provide service at the expense of their education. Another reason for the low attendance could be the fact that our study was entirely voluntary. Those undertaking similar trials in the future may wish to make attendance mandatory. The trial's generalisabilty may be is limited in that the educational intervention itself was brief and only looked at a specific teaching area of EBM and systematic reviews.

The trials small sample size may limit its generalisabilty, but it was adequately powered to address our null hypothesis. The trail forms an important foundation for the development, adaptation and evaluation of new computer based learning techniques for wider application in postgraduate and continuing education. This emphasizes the relevance of computer based teaching which can be made available at their convenience.

Our findings have implications for the way in which postgraduate trainees can be taught. Computer based learning has the potential to meet medical training needs and other professions have already started to embrace it in continuing professional education [21]. Our trial suggests that computer based teaching is a viable alternative to lectures, at least for the teaching of EBM. For those who did not attend sessions it would be available at other times, whereas lectures could not be readily repeated. This would be a massive advantage for doctors who are under constant time pressure. Computer based sessions also allow standardisation of teaching between institutions and addresses the difficulty of teaching a large number of students dispersed over different sites. Computer based learning can be made interactive to encourage better deeper learning [22]. The addition of links to material on the web or contained in other files can enhance the learning experience. With such enhancements, computer based teaching may perform even better than lectures, a hypothesis that will no doubt be subject of future research. As continuing medical education becomes more central to doctors' lives, computer based teaching is one of the ways to meet demands for knowledge.

## Conclusion

Our recommendation is that computer based teaching is a ready alternative to lecture based teaching in EBM for postgraduates. We need to conduct further research to explore the wider potential of computer based learning in medical education.

## Competing interests

The author(s) declare that they have no competing interests.

## Authors' contributions

JD carried out the assembly of questionnaires and teaching as well as the recording of the web based teaching sessions. KK carried out assembly of the questions and validated the teaching content. AC helped assemble questionnaires and advised on randomisation techniques. EC recorded the web based teaching and assisted JD in teaching the sessions. JZ analyzed all the data and performed statistical testing as well as advising on statistical techniques. DD providing equipment for the recording of sessions as well as IT support. All authors read and approved the manuscript.

## Pre-publication history

The pre-publication history for this paper can be accessed here:

http://www.biomedcentral.com/1472-6920/7/23/prepub

## Supplementary Material

Additional file 1 Berlin and Fresno questionnaire, web addresses. This file provides web links to the Berlin and Fresno evidence based medicine questionnaires. Click here for file
