# Supplementary material for: Computer-based teaching is as good as face to face lecture-based teaching of evidence based medicine: a randomised controlled trial
Source: BMC Med Educ. 2007 Jul 20;7:23. doi: 10.1186/1472-6920-7-23 (PMC3225809; doi:10.1186/1472-6920-7-23)
Supplement: Additional file 1 — Berlin and Fresno questionnaire, web addresses. This file provides web links to the Berlin and Fresno evidence based medicine questionnaires. [file 1472-6920-7-23-S1.doc]

**Appendix 1:**

**Berlin questionnaire:** [**http://www.acsu.buffalo.edu/~hjs/SetA-electronicdfd.pdf**](http://www.acsu.buffalo.edu/~hjs/SetA-electronicdfd.pdf)

**Fresno questionnaire:** [**http://www.bmj.com/cgi/content/full/326/7384/319/DC1**](http://www.bmj.com/cgi/content/full/326/7384/319/DC1)
